# *Stigmellamultispicata* Rociene. & Stonis, an Asian leafminer on Siberian elm, now widespread in eastern North America (Lepidoptera, Nepticulidae)

**DOI:** 10.3897/zookeys.784.27296

**Published:** 2018-09-12

**Authors:** Erik J. van Nieukerken, Daniel Owen Gilrein, Charles S. Eiseman

**Affiliations:** 1 Naturalis Biodiversity Center, PO Box 9557, NL-2300 RA Leiden, The Netherlands Naturalis Biodiversity Center Leiden Netherlands; 2 Cornell Cooperative Extension of Suffolk County, Entomology, Riverhead, New York, USA Cornell Cooperative Extension of Suffolk County Riverhead United States of America; 3 276 Old Wendell Rd., Northfield, MA 01360, USA Unaffiliated Northfield United States of America

**Keywords:** Canada, China, DNA barcoding, invasive, key, pest species, *
Stigmella ulmivora
*, *
Ulmus pumila
*, United States

## Abstract

*Stigmellamultispicata* Rocienė & Stonis, 2014, previously known from the single male holotype from Primorye, Russia, is reported as a new invasive species mining leaves of Siberian elm, *Ulmuspumila* L., in eastern North America. Both adults and leafmines have been reported from many sites as unidentified Nepticulidae since 2010. Crucial for the identification was a match of the DNA barcode of a single larva collected on *Ulmuspumila* in Beijing with adults from North America. The single larva constitutes a new record for China. *Stigmellamultispicata* is closely related to the European *S.ulmivora* (Fologne, 1860), feeding likewise on *Ulmus*, but differs in details of external morphology and genitalia, particularly in the female, where *S.multispicata* has a remarkable elongated narrow ovipositor, suitable for oviposition in underside hairy leaf vein axils, where all mines start. In North America *S.multispicata* is the only *Ulmus*-feeding nepticulid with green larvae. Currently the species is known from USA: Illinois, Indiana, Iowa, Maryland, Massachusetts, Minnesota, New York, Ohio, Tennessee, Wisconsin, and Canada: Ontario and Québec. In Sagaponack, on Long Island, New York, larvae have been reported to occur en masse on Siberian elms from at least two sites. The current distribution could be reconstructed thanks also to many online photographs from observation websites. The species is redescribed, with the first descriptions of female, larva, and leafmine, and compared with *S.ulmivora*, which is fully redescribed. The two native North American nepticulid *Ulmus* leafminers, *S.apicialbella* (Chambers, 1873) and *Ectoedemiaulmella* (Braun, 1912), are diagnosed and new provincial and state records are provided. A key to linear mines on *Ulmus* in North America is provided. We suspect that trade of live plants through nurseries played a role in the sudden spread of this invasive species.

## Introduction

The North American insect fauna has been enriched by an influx of numerous alien species. Most invasions in temperate North America originate in Europe, far fewer in Asia (Mattson et al. 1994, 2007). Among phytophagous insects on woody plants, the number reported in 2007 as arriving from Europe is 310 species, compared with only 77 coming from Asia (Mattson et al. 2007). The increasing global travel and transport of goods results in an ever-growing number of established alien insect species, estimated to be more than 40 over a five-year period (Work et al. 2005). While the number and proportion of non-native Lepidoptera is not as large as in some other insect groups, around 250 of the 5431 named Canadian and Alaskan species (4.6%) are thought to be introduced (Pohl et al. 2018). Thanks to DNA barcoding, previously overlooked introductions have been recognized, particularly in taxonomically poorly studied groups (Landry et al. 2013). Here we report another case of a lepidopterous insect introduced from Asia, where DNA barcoding played an essential role in the identification.

Beginning around 2010, James Vargo found numerous specimens of an unknown *Stigmella* species that he had not seen before, while light collecting on his property in Indiana, USA. Material was sent to specialists Don Davis and David Wagner, neither of whom recognized the moths as belonging to a known American species. EJvN got some of these specimens when borrowing unidentified Nepticulidae from the Mississippi Entomological Museum in 2010, and also failed to name them. A near match of the DNA barcode with a larva collected in 2013 from a leafmine on *Ulmus* in Beijing, China, was striking, but initially regarded as coincidental.

In October 2015, DOG received a sample of green nepticulid larvae from Ms. Lee Foster in Sagaponack on Long Island, Suffolk County, New York, that were descending en masse on silk strands from Siberian elm (*Ulmuspumila* L.) (Figs 21–25). Since the two known North American Nepticulidae on elm (see below) have yellow larvae, he contacted EJvN for identification. It soon was clear by comparing DNA barcodes and the emerging moths that these were the same species as the one found in Indiana. The property in Indiana also has several old Siberian elm trees, but leafmines were not seen until 2018 (J. Vargo, pers. comm. to EJvN). Meanwhile, more records with the same DNA barcode turned up in the BOLD database (Ratnasingham and Hebert 2007). The match with the Chinese elm miner now made more sense, and the male genitalia and external features appeared to match the illustrations of the recently described *Stigmellamultispicata* Rocienė & Stonis, 2014, based on a single male holotype from Primorye, Russia (Stonis and Rocienė 2014). It was therefore likely that we were dealing with a recent invasion. Also, several online photos of leafmines and adults, including on BugGuide (2018) and iNaturalist (2018), could now be matched with *S.multispicata* (Table 1).

**Table 1.** Online photographs of adults and leafmines of *Stigmellamultispicata.* Identifications by E.J. van Nieukerken. All leafmines on *Ulmuspumila*.

Stage	State	County/City	Locality	Date	Observer	url
**Canada**						
1 adult *	Ontario	Toronto	Yarmouth Gardens	26.vii.2015	D. Beadle	https://www.inaturalist.org/observations/9482752
1 adult	Ontario	Toronto	Yarmouth Gardens	18.vii.2016	D. Beadle	https://www.inaturalist.org/observations/9542202
**United States**						
1♀	Illinois	Cook	Skokie, Balaban House	24.viii.2017	John & Jane Balaban	https://bugguide.net/node/view/1439557
ca 8 mines	Illinois	Lisle	The Morton Arboretum	4.x.2010	Bruce J. Marlin	http://www.cirrusimage.com/tree_Siberian_Elm.htm
ca. 3 young mines	Illinois	Urbana	residential area	4.ix.2010	John Hilty	http://www.illinoiswildflowers.info/trees/plants/sb_elm.html
3 mines	Iowa	Shelby	south of Elk Horn	19.x.2016	M.J. Hatfield	https://bugguide.net/node/view/1362960
1♂	Iowa	Winneshiek	100 Acre Wood	27.vii.2014	M.J. Hatfield	https://bugguide.net/node/view/966519
1♂ **	Iowa	Winneshiek	Plymouth Rock	26.ix.2014	M.J. Hatfield	https://bugguide.net/node/view/1028011
old mines***	Maryland	Baltimore City	Herring Run Park	15.x.2013	Thomas Wilson	https://bugguide.net/node/view/906301
1 adult	Minnesota	Hennepin	Minneapolis	19.viii.2013	Bill Johnson	https://bugguide.net/node/view/813155
1 adult	Ohio	Pickaway	Orient	7.viii.2012	Gregory Raterman	https://bugguide.net/node/view/686805
1 adult	Tennessee	Davidson	Nashville	25.vii.2010	Steven Loftin	https://bugguide.net/node/view/433176
1 adult	Wisconsin	Dane	Cross Plains	4.ix.2010	Ilona L.	https://bugguide.net/node/view/451465.

* The single photographed adult was said to be part of a “mini invasion” at a light on this night.** This specimen was sent to the authors and is also listed under material examined.*** According to the observer the tree was either *Ulmusparvifolia* or *pumila*, we think it is *U.pumila*.

The Nepticulidae (pygmy moths) of North America were revised almost 40 years ago (Wilkinson 1979, 1981, Wilkinson and Scoble 1979, Wilkinson and Newton 1981; Newton and Wilkinson 1982), but these studies were based on the relatively poor material then available in some museum collections only; no new fieldwork was carried out, and the publications are hampered by the lack of good color illustrations of moths and leafmines. In recent years, extensive collecting and genetic analyses have shown that the nepticulid fauna of North America is much richer than previously thought and also that more revisionary work of old types is needed. The first results of this research were summarized in the recent catalogues of global and Canadian Nepticulidae (van Nieukerken et al. 2016, van Nieukerken 2018). Also, a revival of leafmine studies, partly made possible through online resources (BugGuide 2018, Moth Photographers Group 2018) resulted in an influx of new distribution records.

The Nepticulidae of East Asia are best known from Far East Russia, especially the Primorye region, by studies of J. Stonis (formerly R. Puplesis) and his students (Rocienė and Stonis 2013, Stonis and Rocienė 2013). The study on Chinese nepticulids is still in its infancy [two papers deal with species feeding on Fagaceae (van Nieukerken and Liu 2000, Stonis et al. 2013)] whereas studies on the Japanese fauna are now progressing (Kemperman et al. 1985, Hirano 2013, 2014, Yagi and Hirowatari 2017).

We will here redescribe *Stigmellamultispicata*, compare it with the European *S.ulmivora* (Fologne, 1860) and the other North American species of Nepticulidae feeding on *Ulmus*, and discuss the probability of its invasion from Asia into North America.

## Material and methods

### Material

The material of *S.multispicata* from the United States and Canada originates from several sources; the few adult specimens from Canada were collected during the School Malaise Trap Program (Steinke et al. 2017). Leafmines in China were collected by EJvN during a collaboration between groups in The Netherlands and China in the 1980s (van Driel and van Nieukerken 1985, van Nieukerken and Liu 2000) and again during a 2013 visit of EJvN to Beijing and Tianjin. More data were obtained from internet searches and barcoded specimens on BOLD (Ratnasingham and Hebert 2007).

Material of the other species discussed here mostly originates from the collections of the Naturalis Biodiversity Center, Leiden, Netherlands. The Material examined section provides only the basic locality data, all details on registration numbers, genitalia slides, collectors etc. are provided in the Suppl. material 1.

### Abbreviations for depositories, etc.

**BIN** Barcode Index Number (Ratnasingham and Hebert 2013)

**BIOUG** Biodiversity Institute of Ontario, University of Guelph, Canada

**BOLD** Barcode of Life Data Systems (http://www.barcodinglife.com/)

**CSEC** C.S. Eiseman Research Collection

**MEM** Mississippi Entomological Museum, Starkville, Mississippi, United States

**RMNH**Naturalis Biodiversity Center, Zoological collections, Leiden, The Netherlands

**USNM** National Museum of Natural History, Smithsonian Institution, Washington DC, United States

**ZIN**Zoological Institute, Russian Academy of Sciences, St. Petersburg, Russia

### Methods

For the collection of leafmines in China see van Nieukerken and Liu (2000). Since the collecting of adults and mines in North America was done by several different persons, there are no general methods to describe here.

Genitalia were prepared according to our standard procedures, usually including DNA extraction, which were described earlier in detail (van Nieukerken 1985, van Nieukerken et al. 2010).

Measurements of genitalia were obtained from digital images, using calibrated scaling in the Zeiss AxioVision software; we used a 20× objective for male genitalia and 10× or 20× for female genitalia. Capsule length was measured from vinculum to middle of uncus; valva length from tip of posterior process to ventral edge, excluding the sublateral process; phallus length was measured along the sclerotized tube, from tip, excluding any protruding vesica parts. Total bursa length includes all of the internal genitalia from cloaca to anterior edge of bursa; apophyses were measured from abdominal tip to anterior tip of apophyses. Genitalia measurements are rounded off to the nearest 5 μm. Forewing length was measured from tip of fringe to attachment on thorax, with a Zeiss SV11 stereo-microscope at a magnification of 20×. Antennal segment counts include scape and pedicel; they were counted on photographs or directly under the same stereo microscope.

Photographs of moths were made with an AxioCam MRc 5 digital camera attached to a motorized Zeiss SteREO Discovery V12, using the Module Extended Focus, Zeiss AxioVision software, to prepare a picture in full focus from a Z-stack of ca 10 to 40 individual photos. Leafmines were photographed by EJvN with an AxioCam HRc camera on a Zeiss Stemi SV11 stereo-microscope, without extended focus. Photos by CSE were taken either with a Canon EOS Rebel XSi SLR digital camera, and MP-E 65 mm macro lens (Figure 34), or with a Nikon D50 digital camera and AF Micro Nikkor 105mm lens (Figure 39) and with a Macro Twin Lite MT-24EX flash unit. Genitalia were photographed with an MRc 5 camera on a manually operated Zeiss Axioskop H, without using extended focus. Photographs were edited with Adobe Photoshop ® (various versions), avoiding any change to the real object, but backgrounds are cleaned of excess debris and artifacts by using healing brush and clone tools; tone and contrast are adjusted and some sharpening is used.

Our methodology for DNA barcoding has been described in other papers (van Nieukerken et al. 2012a, 2012c, Doorenweerd et al. 2015, 2016). We present a Neighbor Joining tree, with KP2 distances, of the selected taxa, made with tools provided by BOLD Systems (Ratnasingham and Hebert 2007). The DNA barcode data as used here are given in detail in the public BOLD dataset DS-STMULT (*Stigmellamultispicata* on *Ulmus*, https://doi.org/10.5883/DS-STMULT).

## Taxomomy

### Stigmella multispicata

Taxon classificationAnimaliaLepidopteraNepticulidae

Rocienė & Stonis

Figures 1 , 2 , 5–6 , 7–9 , 12 , 14 , 16 , 18 , 19–25 , 26 , 28 , 29

Stigmella multispicata Rocienė & Stonis in Stonis & Rocienė, 2014: 205. Holotype ♂, Russia, Primorskiy Kray, 20 km E Ussuriysk, Gornotayezhnoe, Biological Station, 8.viii.2011, leg. A. Rocienė, genitalia slide no. AG427 (ZIN) [not examined].
Stigmella multispicata ; van Nieukerken et al. 2016: 106, 173 [moved to S.ulmivora group].

#### Diagnosis.

In North America *S.multispicata* is the only *Stigmella* species with the combination of black frontal tuft, white collar, and single fascia. *Stigmellaquercipulchella* (Chambers, 1882) is relatively similar, but has an additional silver patch at tornus, is slightly larger and has more strongly purple reflections across the forewings. This combination of characters is also diagnostic in East Asia, but there remains a possibility that similar species will be discovered. From the closely related European *S.ulmivora* it differs by the white collar (dark in *ulmivora*) and the entirely dark antennae (those of *S.ulmivora* have the distal 7–8 flagellomeres white); *S.ulmivora* is also slightly larger and has more antennal segments. The female of *S.multispicata* differs from all more or less similar species by the obvious long ovipositor, visible even without dissection.

Male genitalia differ from those of *S.ulmivora* in the shallowly indented uncus and the very short sublateral processes of the transtilla; the female genitalia are easily recognized by the long apophyses; the ductus spermathecae has no spines in contrast to *S.ulmivora*. Some species in the *S.rhamnella* group have superficially similar male genitalia, but they have no juxta, and the moths are externally very different.

*Stigmellamultispicata* leafmines are characterized by the egg placement on the leaf underside in vein axils, larval exit on leaf underside, and green to blue-green larval color.

#### Redescription.

**Figures 1–4.**
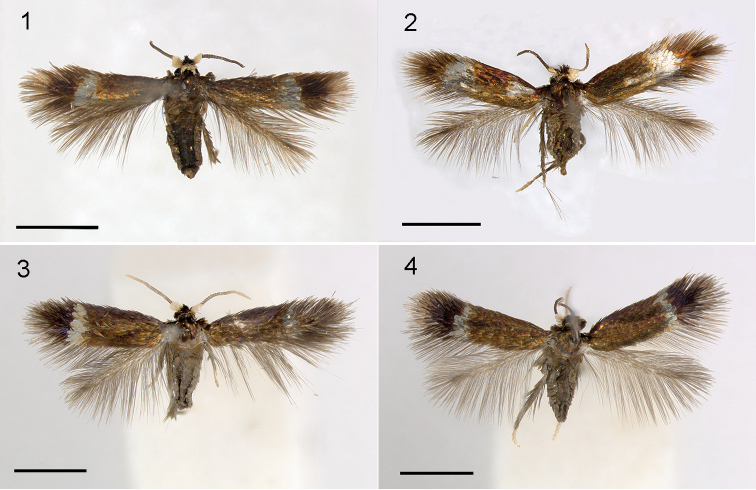
*Stigmella* species, adult habitus. **1***S.multispicata*, male, USA, Indiana, RMNH.INS.24511 **2***S.multispicata*, female, USA, Indiana, 10.viii.2010 **3***S.ulmivora*, male, Netherlands, RMNH.INS.15496 **4***S.ulmivora*, female, Netherlands, RMNH.INS.15497. Scale bars 1 mm. Photographs C. Doorenweerd (1), E.J. van Nieukerken.

**Figures 5–6.**
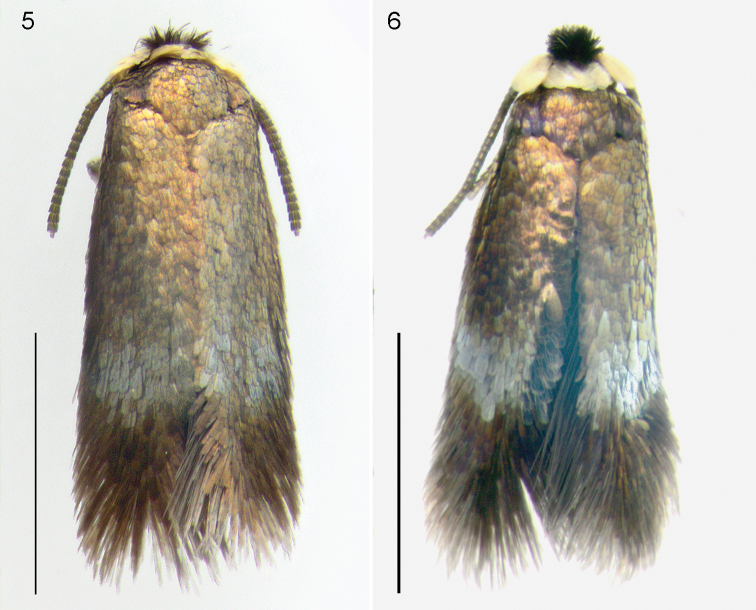
*Stigmellamultispicata*, dead male moths, unmounted, both from USA, Iowa. **5** 26.ix.2014 **6** 27.vii.2014. Scale bars: 1 mm. Photographs M.J. Hatfield.

Male (Figs 1, 5, 6). Forewing length 1.8–1.9 mm (1.8 ± 0.1, 9), wingspan 3.7–4.1 mm. Head: frontal tuft black, collar cream white. Scape cream white. Antenna fuscous, short, with 21–24 segments (22.8 ± 1.1, 5), ratio to forewing length 12–13 segments/mm. Thorax and forewing shining fuscous bronze, in some lights appearing greenish, a silver fascia at 2/3, apex darker fuscous, terminal cilia concolorous, underside dark fuscous. Hindwing grey-brown. Abdomen brown, without visible anal tufts.

Female (Figure 2). Forewing length 1.8–2.2 mm (1.9 ± 0.2, 7), wingspan 4.0–4.3 mm. Antenna with 18–20 segments (18.3 ± 0.8, 6), ratio to forewing length 8–11 segments/mm. Otherwise as male, abdomen with conspicuous long protruding ovipositor, with small anal tufts.

**Figures 7–13.**
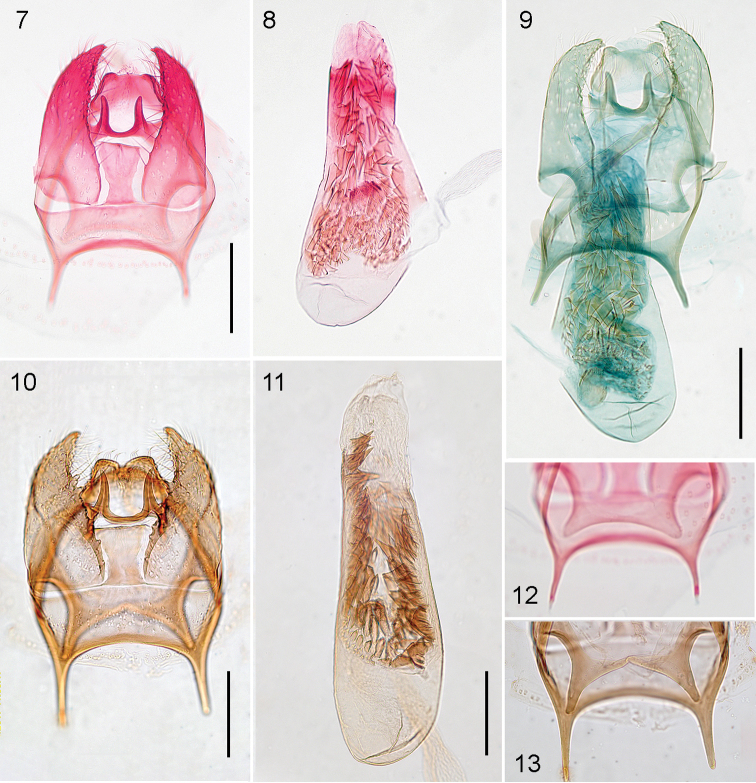
*Stigmella* species, male genitalia in ventral view. **7**, **8**, **12***S.multispicata*, slide EvN4511, RMNH.INS.24511 **9***S.multispicata*, slide JCK8417 **10, 11, 13***S.ulmivora*, slide VU0440, RMNH.INS.20440. **12** and **13** show the difference in transtilla. Scale bars: 100 μm. Photographs E.J. van Nieukerken.

Male genitalia (Figs 7–9, 12). Capsule length 190–235 μm (215.6 ± 23.0, 3), 1.1–1.2× as long as wide. Vinculum anteriorly with pointed and anteriorly protruding lateral corners. Uncus with shallow lobes, widely separated. Gnathos with widely separated posterior processes, running parallel. Valva length 170–185 μm (177.2 ± 5.0, 3), narrow, 2.5–3.0× as long as wide, distally becoming narrower, slightly curved inwards, transtilla with pointed corners, sublateral processes almost absent (Figure 12). Juxta present, haltere-shaped, with triangular point distally. Phallus 320–330 μm (323.4 ± 4.8, 3), 2.3–2.9× as long as wide; vesica with many relatively stout cornuti, varying from long-pointed to broadly triangular, with anterior cornuti smaller.

**Figures 14–18.**
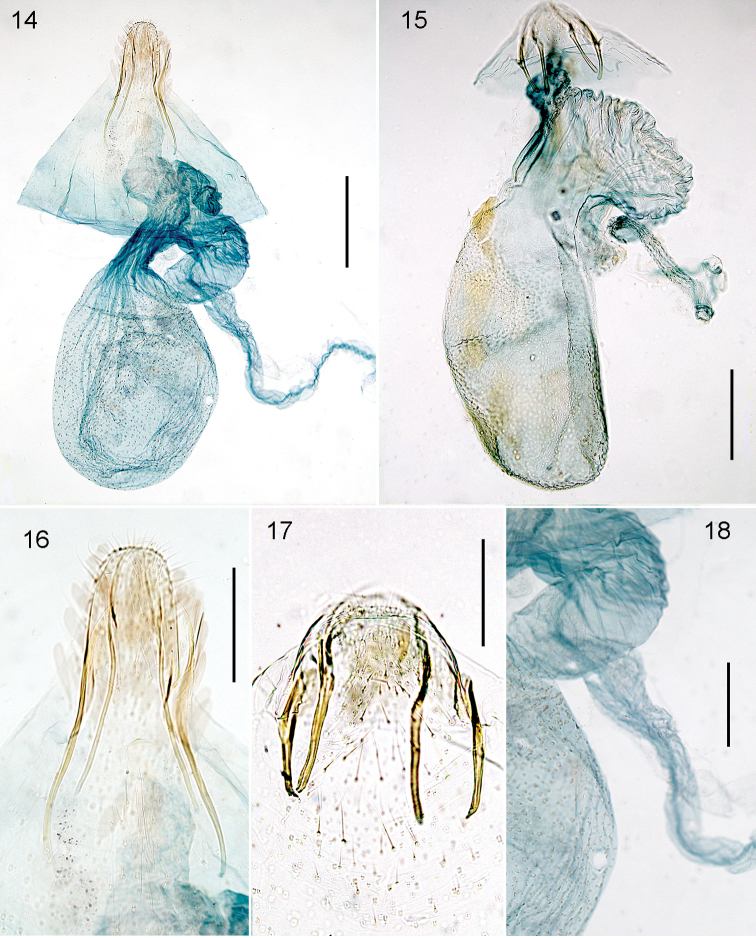
*Stigmella* species, female genitalia. **14**, **16**, **18***S.multispicata*, slide JCK8416 **15***S.ulmivora*, slide VU1638, RMNH.INS.21638 **17***S.ulmivora*, slide VU1769, RMNH.INS.21769. Scale bars: 200 μm (14, 15), 100 μm. Photographs E.J. van Nieukerken.

Female genitalia (Figs 14, 16, 18). No anal papillae; T8 narrow, anterior and posterior apophyses long and narrow, anterior ones longer (ca 290 μm) than posterior (ca 235 μm). Bursa length ca 810 μm; accessory sac strongly curved. Corpus bursae completely covered with relatively distinct pectinations; accessory sac and vestibulum without sclerotizations. Ductus spermathecae originating from accessory sac, with many narrow and indistinct convolutions.

Larva (Figs 23, 25, 26). Head-capsule length 290 μm, width 315 μm.

**Figures 19–25.**
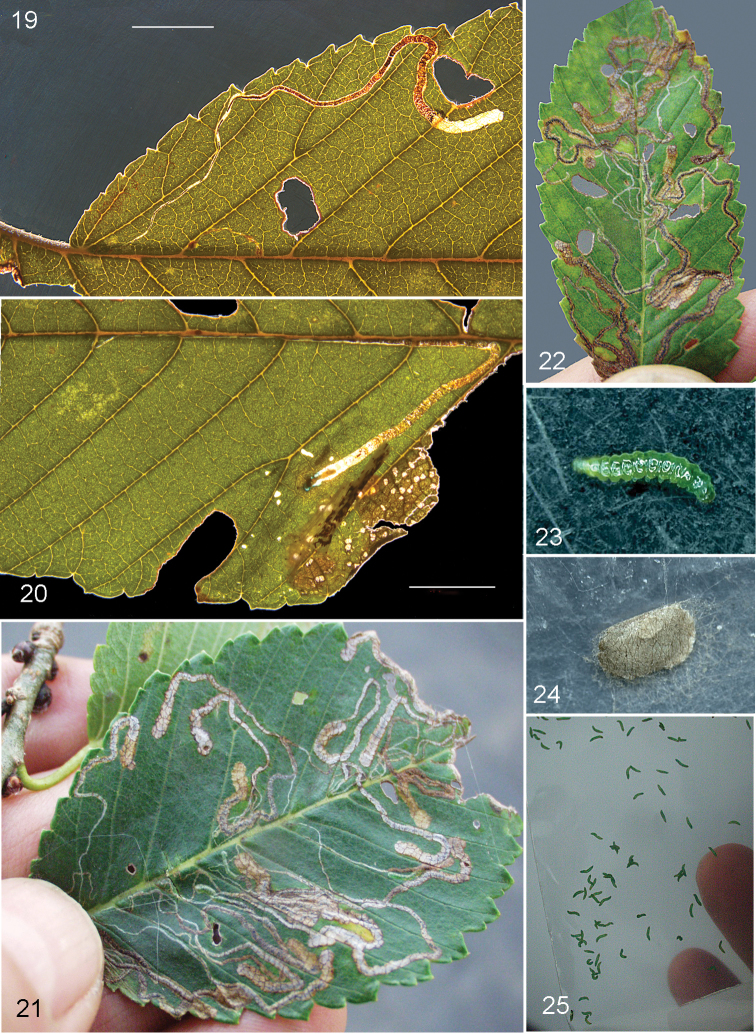
*Stigmellamultispicata*, immature stages and leafmines on *Ulmuspumila*. **19** leafmine of barcoded larva, RMNH.INS.30070. China, Beijing **20** leafmine with dead larva, same locality **21–25** all from NY, Sagaponack, 23.x.2015 and 28.x.2015 (cocoon) **25** showing large number of emerged larvae on plastic sheet. Scale bars: 5 mm. Photographs E.J. van Nieukerken (**19, 20**), D.O. Gilrein (**21–25**).

#### Biology.

*Host plants. Ulmuspumila* L. (Ulmaceae), Siberian elm, a widespread tree in East Asia, cultivated globally in temperate climates, widely planted in North America. Vacated mines presumably representing this species were also collected in China on *Ulmusmacrocarpa* Hance, Large-fruited elm.

*Leafmine* (Figs 19–22). Egg always deposited in vein axils on leaf underside, beneath the trichomes; leafmine a long narrow upper side gallery or corridor mine, running through leaf, usually not along veins and not crossing midrib; slightly curved, but many mines make a U-turn near the end. Frass initially in narrow medial black line, later becoming contorted, brown and almost filling mine. Larval exit on leaf underside.

**Figures 26–27.**
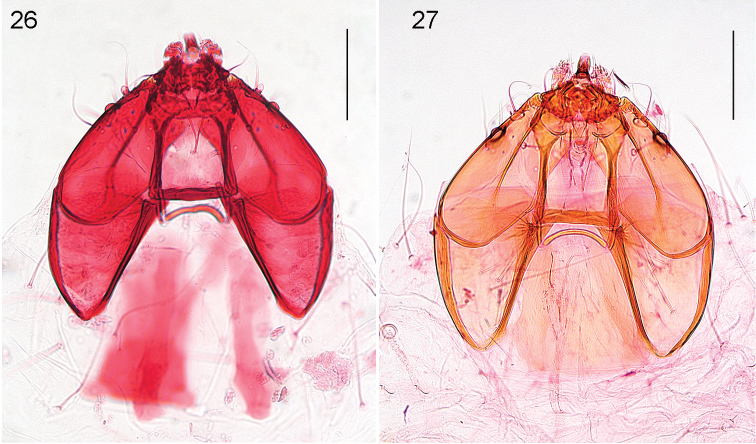
*Stigmella* species, larval headcapsules, dorsal view. **26***S.multispicata*, RMNH.INS.30698 **27***S.ulmivora*, RMNH.INS.18871. Scale bars: 100 μm. Photographs E.J. van Nieukerken.

*Larva* (Figs 23, 25, 26). Bright green to blue-green, probably feeding with venter upwards (analogy with *S.ulmivora*, but not positively observed); head capsule translucent brown. Larvae descending by silken threads, sometimes en masse, spinning a brown cocoon on debris.

*Life history.* Larvae and leafmines found in China in October; in North America larvae were observed from 15 June to mid-July and from 19 October to 6 November. Moths were found on 26 May and from 8 July throughout August to 6 September (with a peak between 10 and 15 August), a few late records from 22 and 26 September and 2 October. Moths reared from October mines emerged in the laboratory between 25 March and 19 May. The species has at least two annual generations, maybe more. Adults are frequently found at light.

#### Distribution

**Figures 28–29.**
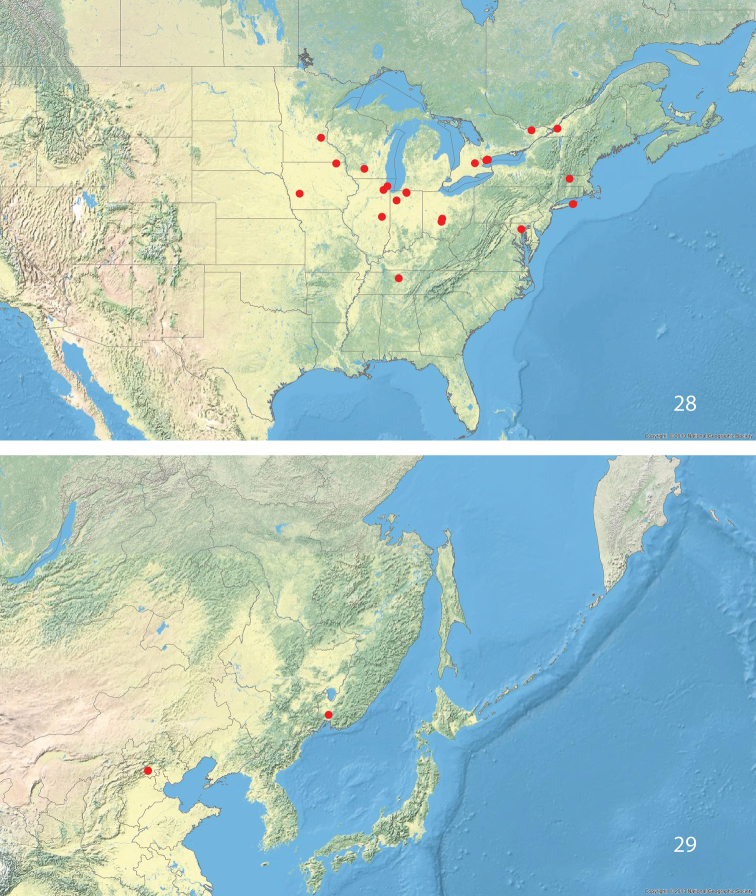
*Stigmellamultispicata*, distribution.

(Figs 28, 29). Presumed to be native in Russia: Primorye and China: Beijing. Almost certainly introduced in North America: Canada: Ontario, Québec; United States: Illinois, Indiana, Iowa, Maryland, Massachusetts, Minnesota, New York, Ohio, Tennessee, Wisconsin. The species has been found in the urban environment, in farmland and in more natural habitats. Table 1 lists the online photographs that we recognized as representing *S.multispicata*.

#### DNA barcodes.

All eight barcodes belong to BINBOLD:ACP7362. All North American barcodes are 100% identical; the single Chinese one differs in 11 basepairs (1.7%). The nearest neighbor, at 6.3%, is *Stigmellaulmivora* (Figure 44).

#### Remarks.

The extensive collections of Chinese microlepidoptera of Nankai University were searched in vain for this species (Li Houhun, personal communication to EvN). It is possible that specimens can be found in other collections in China, such as the Zoological Institute in Beijing.

#### Material examined.

19♂, 26♀, 3 sex undetermined, 5 larvae, mines. **China**: 1 larva (green, dried out, destructively extracted for DNA), China, Beijing, Beijng Botanical garden – Wofosi, E.J. van Nieukerken & S. Richter, 17.x.2013, EvN no 2013117–M, *Ulmuspumila*, N40.00417, E116.20419, 108 m, RMNH.INS.30070; 1 mine from which previous larva was taken, RMNH.INS.43922; 1 mine with dead larva, same data, EvN no 2013117–H, RMNH.INS.43923 (all RMNH).

**Canada**: 1♂,1♀ (in ethanol 96%), Ontario, Toronto, Etobicoke School of the Arts, EQP–CLL–602, Brad Schumacher, 22–28.ix.2014, GMP#05745, Malaise trap, N43.631, W79.504, 109 m, BIOUG16150–E04, BIOUG16150–E05 (BIOUG); 1 vacated leafmine, Ontario, Ottawa, Bayview Rd., E.J. van Nieukerken, 12.vii.2018, *U.pumila*, amidst 100’s of mines of *Orchestessteppensis*, EvN no 2018080H, N45.40819, W75.72474, RMNH.INS.45003 (RMNH); 4 vacated mines, Québec, Montreal, Old Montreal, Avenue de l’Hotel de Ville, E.J. van Nieukerken, 1.viii.2018, *U.pumila*, EvN no 2018101-H, N45.50894, W73.55604, RMNH.INS.45004 (RMNH).

**United States**: 1♀, Indiana, St. Joseph Co., J. Vargo, 26.v.2010, N 41.37’46.2”–W 86.08’13.9”, [N41.62950, W86.13719] (USNM); 1♂, same locality, 2.viii.2010 (USNM); 5♂, 4♀, same locality, 10.viii.2010, genitalia slides JCK8416, JCK8417 (♂), JCK8617, RMNH.INS.15499 (RMNH, MEM, USNM); 1♂, same locality, 13.viii.2010, genitalia slide EvN4511, RMNH.INS.24511 (RMNH); 1♂, same locality, 11.viii.2010 (MEM); 1♀, same locality, 13.viii.2010 (USNM); 5♂, 16♀, same locality, 15.viii.2010 (MEM, RMNH); 1♀, same locality, 28.viii.2010 (MEM); 1♂, 2♀, same locality, 6.ix.2010 (USNM); 1♂ (abdomen missing), Indiana, Pulaski Co., Jasper-Pulaski FWA, J. Vargo, 4.viii.2010, 41 09’ 31.0”N 086 58’ 42.6”W [N41.15861,W86.9785] (USNM); 1♂, Iowa, Winneshiek Co., Plymouth Rock, Black light in a planted prairie near woodlands along the Upper Iowa River, M.J. Hatfield, 26.ix.2014, N43.4376, W92.0041, Genitalia slide EvN5052, RMNH.INS.25052 (RMNH); 14 leafmines (13 vacated, 1 with dead larva), Massachusetts, Franklin Co., Sunderland, N42.498380, W72.544853, C.S. Eiseman, 22.vii.2018, *U.pumila* (CSEC); 4 larvae (in Tissue collection, ethanol 96%, –80°, 1 preparation), New York, Suffolk Co., Sagaponack, Ms. Lee Foster, 21.x.2015, *U.pumila*, N40.93, W72.28, RMNH.INS.30697, RMNH.INS.30698 (extracted), larval preparation RMNH.INS.30698.P, RMNH.INS.30699, RMNH.INS.30700 (RMNH); 3 adults (in capsule), same data, emerged 25.iii & 19.v.2016, (RMNH); 1♂, Ohio, Franklin Co., Hillard, D.J. Shetlar, 21.vii. 2016, N40.007, W83.1738, Genitalia slide EvN4901, RMNH.INS.24901; 1♂, same locality, 1.ix.2016, RMNH.INS.15533 (RMNH).

#### Tentative ID, most likely this species.

**China**: 4 tenanted mines (rearing failed), Beijing, Xiangshan, Wofosi and botanical garden, E.J. van Nieukerken & J.W. van Driel, 1.x.1984, EvN no 18–1–1K, Hills with deciduous shrub and low trees, *U.macrocarpa*, N39.983, E116.2, 100–500 m, RMNH.INS.44328; 1 vacated mine, same data, EvN no 18–1–1H, RMNH.INS.44330.

#### Other data, material not examined.

**Canada** (Data from BOLD, barcode identification): 1 adult, Ontario, Waterloo region, Kitchener, Crestview Public School, EQP-CLL-863, Sherrie Cochrane, 2.x.2015, GMP#08378, N43.454, W80.44, 334m, BIOUG25491–E12 (BIOUG); 1 adult, Ontario, Toronto, Eastdale CI, EQP–CLL–605, David Servos, 2.x.2015, GMP#08428, N43.666, W79.349, 89, BIOUG25505–D03 (BIOUG).

**United States** (Observations, personal communications to authors): 4 larvae/mines, Indiana, St. Joseph Co., J. Vargo, 15–29.vi.2018; 120 adults, St. Joseph Co., Mishawaka, J. Vargo, 8.vii.2018, light trap; larvae descending en masse from trees, New York, Suffolk Co., Sagaponack, Ms. Lee Foster, 21.x.2015, *U.pumila*, N40.93, W72.28; larvae still present, same locality, 8.xi.2015; larvae, same locality, Mike Harmon, 16.vii.2016; larvae, same locality, 19.x.2016; larvae descending en masse from trees, New York, Suffolk Co., Wainscott, 3.xi.2015, Mike Harmon, *U.pumila*, N40.94, W72.24.

### Stigmella ulmivora

Taxon classificationAnimaliaLepidopteraNepticulidae

(Fologne) Beirne

Figures 3 , 4 , 10 , 11 , 13 , 15 , 17 , 27 , 30–33

Nepticula ulmivora Fologne, 1860: 199. Syntypes, Belgium, Brussels region, reared from Ulmus, 1859, emerged 1860, Fologne [probably lost].
Stigmella ulmivora ; Beirne 1945: 199 [recombination]; van Nieukerken et al. 2016: 105 [full synonymy].

#### Diagnosis.

*Stigmellaulmivora* can be separated from *S.multispicata* by the slightly larger size, the dark collar, and the antennae with the terminal 7–8 flagellomeres white. In Europe and North America there are no other *Stigmella* species with the same combination of characters. The male genitalia are very similar to those of *S.multispicata*, but have a deeper indentation in the uncus, and longer and more distinct sublateral processes of the transtilla. The female differs by the blunt ovipositor and the spiny ductus spermathecae.

Leafmines differ from those of *S.multispicata* by the egg position not being in vein axils; in Europe mines are inseparable from those of *S.ulmiphaga* (Preissecker, 1942). Due to the variability of mines of *S.ulmivora*, they sometimes are difficult to separate from those of *S.lemniscella* (Zeller, 1839), from which the yellow larva emerges through the leaf upper side, not the underside as in *S.ulmivora*.

#### Redescription.

Male (Figure 3). Forewing length 2.1–2.5 mm (2.3 ± 0.1, 10), wingspan 4.6–5.2 mm. Head: frontal tuft black, collar cream white. Scape cream white. Antenna fuscous, terminal 7–8 flagellomeres completely white, with 25–29 segments (26.7 ± 1.6, 10), ratio to forewing length 10–13 segments/mm (11.6 ± 0.9, 10). Thorax and forewing shining fuscous bronze, a silver fascia at 2/3, apex darker fuscous, terminal cilia concolorous, underside dark fuscous. Hindwing grey-brown. Abdomen brown, no visible anal tufts.

Female (Figure 4). Forewing length 1.9–2.6 mm (2.3 ± 0.2, 10), wingspan 4.2–5.4 mm. Antenna with 19–23 segments (21.0 ± 1.2, 8), ratio to forewing length 8–11 segments/mm (9.3 ± 1.0, 8). Otherwise as male, abdomen with conspicuous long protruding ovipositor, with small anal tufts.

Male genitalia (Figs 10, 11, 13). Capsule length 190–210 μm (203.8 ± 10.2, 4), ca 0.9× as long as wide. Vinculum anteriorly with pointed and anteriorly protruding lateral corners. Uncus distinctly bilobed, lobes adjacent. Gnathos with widely separated posterior processes, running parallel. Valva length 180–185 μm (182.4 ± 0.7, 4), rather narrow, 2.0–2.4× as long as wide, distally becoming narrower, slightly curved inwards, transtilla with pointed distinct sublateral processes (Figure 13). Juxta present, haltere-shaped. Phallus 275–440 μm (351.1 ± 68.4, 4), 2.0–3.3× as long as wide; vesica with many relatively stout cornuti, varying from long and pointed to broadly triangular, with anterior cornuti smaller.

Female genitalia (Figs 15, 17). No anal papillae; T8 rounded, not elongated, anterior and posterior apophyses short, almost equal in length, anterior ones ca 170–210 μm, posterior ca 185–200 μm. Bursa length ca 770–930 μm; accessory sac strongly curved. Corpus bursae completely covered with relatively distinct pectinations; accessory sac and vestibulum without sclerotizations. Ductus spermathecae originating from accessory sac, basally wide and covered with many spines, with several narrow and indistinct convolutions.

Larva (Figs 27, 32). Head-capsule (n=2) length 300–310 μm, width 300–315 μm.

#### Biology.

*Host plants. Ulmusminor* Mill., *U.glabra* Huds., *Ulmus* spp. (Ulmaceae). Reared specimens labeled as coming from *Acer* were from cocoons found on trunks of that tree; therefore this cannot be considered a host record.

**Figures 30–33.**
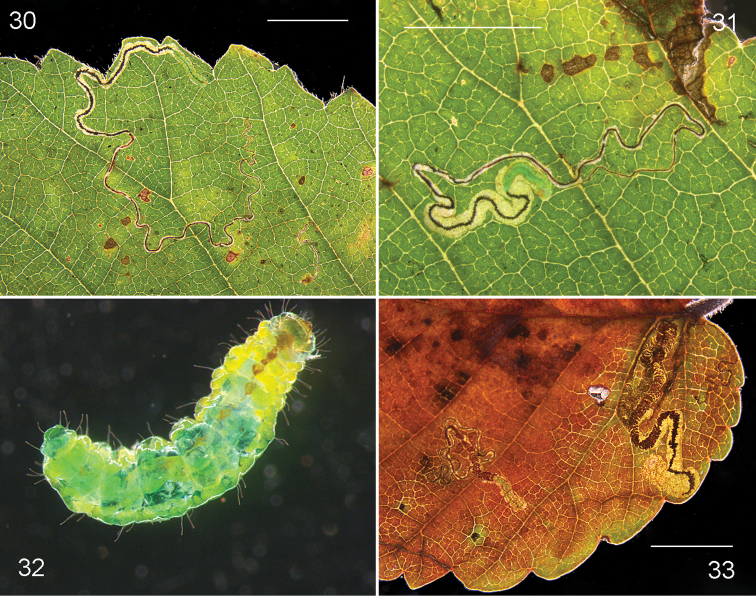
*Stigmellaulmivora*, leafmines and larvae on *Ulmusminor*, Greece. **30–32** Akhaia, Strofilia, 4.xi.2011, larva RMNH.INS.29046 **33** Ilía, Olympia, 5.xi.2011. Scale bars: 5 mm. Photographs E.J. van Nieukerken.

*Leafmine* (Figs 30–33). Egg on leaf underside, against a vein. Mine a highly variable gallery, ranging from short and filled with dense frass in thick leaves (usually in the sun) to long and narrow, often partially following a vein, with frass either linear or becoming contorted, mines sometimes much winding. Larval exit on leaf underside.

*Larva* (Figure 32). Bright green, feeding with venter upwards; head capsule translucent brown. Larvae descending by silken threads, spinning a brown cocoon on debris or on tree trunks.

*Life history.* Bivoltine, or possibly partially univoltine in northern parts of Europe. Larvae in June to early July, again in August to November. Adults recorded from May (a single April record) to early July and again in August.

#### Distribution.

Widespread throughout Europe, east to the Volga region in Russia (Johansson and Nielsen 1990, van Nieukerken 2017). The species occurs both in natural habitats and on trees in cities, often in large numbers (EJvN, personal observations).

#### DNA barcodes.

We have 13 barcodes from across Europe, all belonging to BINBOLD:AAI0023, with some variation, a maximum distance for the Greek barcodes of 2.41% to the rest. The nearest neighbor, at 6.31%, is *Stigmellamultispicata* (Figure 44).

#### Material examined.

**Adults**: 25♂, 50♀. **Croatia**: 1♂, Krk, Kampelje, 17.viii.2001; 1♀, Krk, Mt. Hlam, loc. Branusine, 15.viii.2012. **Germany**: 1♀, Berlin; 1♂, Thüringen, Bad Blankenburg, Muschelkalk, 1.vii.1986; 1♀, Thüringen, Bad Blankenburg, Schwarzatal, 5.vii.1986. **Italy**: 1♂, Cuneo, Entracque, ca 1 km SE, Il Bosco, 16.viii.2007, la on *Ulmus*; 1♂, 1♀, same locality, 13.x.2008, la on *Ulmus*; 1♀, Latina, Monti Aurunci, 4 km NW Castelforte, 22–23.vi.1969. **Netherlands**: 2♂, 2♀, Gelderland, Wageningen, *Ulmus*, e.l. 19.v.1977; 1♀, ibidem, larva x.1989, *Ulmus*; 2♂, 3♀, Noord-Brabant, Breda, v.1877; 1♀, ibidem, e.l. 10.vi.1877, “acer pseudop.”; 2♂, 1♀, ibidem, e.l. 25.vi.; 1♀, 3 damaged adults, ibidem, v.1878; 1♀, Noord-Holland, Amsterdam, 2.viii.1937; 1♂, Noord-Holland, Amsterdam, Koloniaal instituut, 7.viii.1937; 1♀, Noord-Holland, Amsterdam, *Ulmus*, e.p. 30.vi.1929; 1♀, ibidem, *Ulmus* e.l. 27.iv.1942; 5♀, ibidem, *Ulmus* e.l. 29.vii–2.viii.1943; 1♀, Noord-Holland, Amsterdam N.W., *Ulmus*, 16.viii.1942; 1♂, 4♀, ibidem, *Ulmus* e.l. 2–14.viii.1947; 1♀, ibidem, *Ulmus* e.l. 6.vi.1948; 1♀, ibidem, *Ulmus* e.l. 11.vi.1948; 1♀, Noord-Holland, Bussum, 16.iv.1934; 1♂, Noord-Holland, Castricum, 15.viii.1979, *Ulmus* e.l. 13.vi.1980; 6♂, 9♀, Noord-Holland, Hilversum, *Ulmus* e.l. 4–24.vi.1943; 4♀, ibidem, *Ulmus* e.l. 17.v.–6.vi.1945; 1♀, Noord-Holland, Overveen, 7.vi.1929; 1♂, ibidem, 1.vii.1929; 2♀, Zuid-Holland, Den Haag, *Ulmus* e.l. 9.v–10.vi.1865; 1♀, Zuid-Holland, s Gravenhage, 29.v; 1♀, Zuid-Holland, Lexmond, 3.viii.1999; 1♂, Zuid-Holland, Rottm. [Rotterdam], e.p. 22.vi.1901; 1♂, ibidem, e.l. 24.vi.1864; 1♂, ibidem, e.l. 13.vii. 1870; 1♂, ibidem, 21.vi.1877; 1♀, ibidem, pupa in iv, e.p. 21.vii.1879; 1♀, ibidem, 27.v.1877. **Poland**: 1♂, Silesia, Wroclaw (Breslau), e.l. iii.1882.

**Leafmines and larvae**. When no hostplant is given, read *Ulmus* sp. – **Czech Republic**: 1 mine, Moravia, Lednice, 3 km SW, forest near lake, 3.x.1992. **France**: 4 larvae, vacated mines, Alpes-Maritimes, Saorge, 0.8 km SW, vallée de Roya, 9.x.2008, *U.minor*; 1 vacated mine, Alpes-Maritimes, Tende, ca 1 km S, E. slope, 10.x.2008, *U.glabra*; mines, Bouches-du-Rhône, Aix-en-Provence, Parc Jourdan, 12–16.x.1983; several vacated mines, Cher, Villeneuve-sur-Cher, 30.vii.2009, *U.minor*; vacated mines, Drôme, Beaurières, 4 km W: Marais des Boulignons, 21.viii.2002; 1 vacated mine, Eure, Le Marais Vernier, la Vallée, 5.x.2017; 2 vacated mines, Finistère, Presqu’île de Crozon, ca 4 km E Crozon, l’Aber, 12.vii.2006; 1 vacated mine, Haut-Rhin, Colmar, 3 km SW, Le Neuland, 26.ix.2002; 1 larva, 4 mines, Haut-Rhin, Lapoutroie, La Bohle, 21.x.2002, *U.glabra*; 3 vacated mines, Indre-et-Loire, Huismes, N of Contebault, along C17, 8.x.2017; several vacated mines, Lozère, Marjoab, 2.5 km SW Meyrueis, 22.vii.2009, *U.minor*; 3 vacated mines, Lozère, Barre-des-Cévennes, 26.vii.2009, *U.minor*; vacated mines, Pyrénées-Orientales, Port-Vendres, near railway station, 28.vii.1982. **Germany**: 1 vacated mine, Brandenburg, Erkner, 9–11.ix.2007; several vacated mines, Saarland, Neunkirchen, Zoo, 30.ix.2004; 1 vacated mine, Sachsen-Anhalt, Dornburg, 2 km S, along Elbe, 19.vii.2014. **Greece**: 2 larvae, many mines, Akhaia, Strofilia, S Kalogria, 4.xi.2011, *U.minor*; 6 larvae, several mines, Ilía, Olympia, archeological site, 5.xi.2011, *U.minor*. **Italy**: 1 vacated mine, Bolzano, Vinschgau, Prad an Stilfser Joch, Suldenbach banks, 27.vii.2005; 1 larva, many mines, Cuneo, Entracque, ca 1 km SE, Il Bosco, 16.viii.2007; several mines, Cuneo, Entracque, ca 1 km SE, Il Bosco, 13.x.2008; 3 vacated mines, Roma, Trevignano Romano, 17.ix.2005. **Monaco**: 3 vacated mines, Monaco Ville, E slope of Palais Principier, 9.viii.2007. **Netherlands**: mines, Gelderland, Winterswijk, Bekendelle, along Slinge, 1.x.1979; 1 larva, mines, Gelderland, Nijmegen, Winkelsteeg, 11.x.2008; mines, Limburg, Eys, railway near Piepert, 11.ix.1979; mines, Limburg, Thorn, klooster Bethanien, 24.ix.1979; mines, Limburg, Gulpen, NW, holle weg, 8.x.1979; 1 mine, Limburg, Thorn: Baarstraat, 3.x.1988; mines, Noord-Holland, Castricum, hedge along road, 15.viii.1979; mines, Noord-Holland, Castricum, hedge along road, 15.viii.1979; mines, Noord-Holland, Amstelveen, Beneluxbaan, median reserve, 21.ix.1979; several vacated mines, Overijssel, Weerribben, Ossenzijl, Venebosch, 27.viii.2011; several vacated mines, Zeeland, Middelburg N., Brigdamseweg, 2.viii.2009; 1 larva, 1 mine, Zuid-Holland, Den Haag, Waalsdorpervlakte, 7.x.2007; mines, Zuid-Holland, Leiden W, experimental garden University, 5.vii.1979; mines, ibidem, 5.ix.1979; mines, Zuid-Holland, Wassenaar, Meijendel, near Kijfhoek, 18.ix.1979; 1 larva, Zuid-Holland, Oegstgeest, Rhijngeest, 23.ix.1997. **Portugal**: 1 mine, Tras-os-Montes, PN Montesinho, Salgueiros, Vallone das Furnas, 8 km N Vinhais, 30.vii.2001. **Romania**: a few vacated mines, Brasov, Brașov, Mt. Tâmpa, 2.viii.2011. **Sweden**: several vacated mines, Bohuslan, Svenneby, Valön Nature Reserve, 7.viii.2008.

#### North American Ulmus leafminers

##### 

Previously only two Nepticulidae were known to feed on *Ulmus* in North America: *Stigmellaapicialbella* (Chambers, 1873) and *Ectoedemiaulmella* (Braun, 1912). This is a much poorer fauna than the seven European species (van Nieukerken 1986, Puplesis 1994), and in Asia the number is probably still higher, but for several species that are potentially *Ulmus* feeders the hosts are as yet unknown.

Identification of the North American Nepticulidae mines and adults reared from *Ulmus* is straightforward. For convenience we provide a key that distinguishes these from other insects that form partially or entirely linear mines. Primary blotch mines on elm are formed by additional species of Lepidoptera (Coleophoridae: *Coleophora*; Gracillariidae: *Cameraria*, *Phyllonorycter*), Coleoptera (Buprestidae: *Brachys*; possibly also Curculionidae: *Tachygonus*), and Hymenoptera (Tenthredinidae: *Fenusa*). For a complete key, see Eiseman (2018).

##### Key to North American *Ulmus* (linear) leafmines

**Table:** 

1	Linear-blotch mine formed in spring; egg inserted in the leaf	**2**
–	Linear or linear-blotch mine formed in summer or fall; egg deposited on the leaf surface	**3**
2	Mine originating at a scar on the underside of the leaf midrib; frass forming a central line in the linear portion; larva with a head capsule, pupating in a globular cocoon within the mine	***Orchestes* spp.** (Coleoptera: Curculionidae)
–	Mine typically originating near the leaf margin, not associated with a scar; frass indistinct in the linear portion; larva without a head capsule, exiting the mine to pupate	***Agromyzaaristata* Malloch, 1915** (Diptera: Agromyzidae)
3	Mine approx. 2 cm long and less than 1 mm wide, mostly following the midrib and one or two lateral veins; frass filling the width of the mine except for the terminal several mm, in which no frass is deposited; larva emerging to feed externally in patches on the lower leaf surface	***Bucculatrix*** (Lepidoptera: Bucculatricidae)
	Note: *Bucculatrixeclecta* Braun, 1963 has been reared from elm but no details of the mine were recorded (Braun 1963). The above description is based on mines of an undetermined species collected on *Ulmusalata* in NC by T. S. Feldman and on *U.pumila* in MA by C. S. Eiseman.	
–	Mine longer than 2 cm and eventually more than 1 mm wide, with extended portions that do not follow veins; frass at least initially forming a narrow central line; larva feeding as a miner throughout its development	**4**
4	Mine distinctly linear throughout its length; egg usually on the lower leaf surface; cocoon never spun within the mine	**5**
–	Mine contorted to form a blotch in the later portion, although parts of the mine may be linear; egg often on the upper leaf surface; cocoon of overwintering generation sometimes spun within the mine (Figs 40–43)	***Ectoedemiaulmella*** (Lepidoptera: Nepticulidae)
5	Egg deposited on either leaf surface, may be next to a vein but not in an axil; larva yellow, exiting through the upper epidermis; on native elms (Figs 36–39)	*** Stigmella apicialbella ***
–	Egg deposited on the lower leaf surface, in a vein axil and beneath the trichomes; larva green, exiting through the lower epidermis; on Siberian elm (Figs 19–22)	*** Stigmella multispicata ***

### Stigmella apicialbella

Taxon classificationAnimaliaLepidopteraNepticulidae

(Chambers) Newton & Wilkinson

Figures 34 , 36–39

Nepticula apicialbella Chambers, 1873: 127.
Stigmella apicialbella : Newton and Wilkinson 1982: 413.

#### Diagnosis.

**Figures 34–35.**
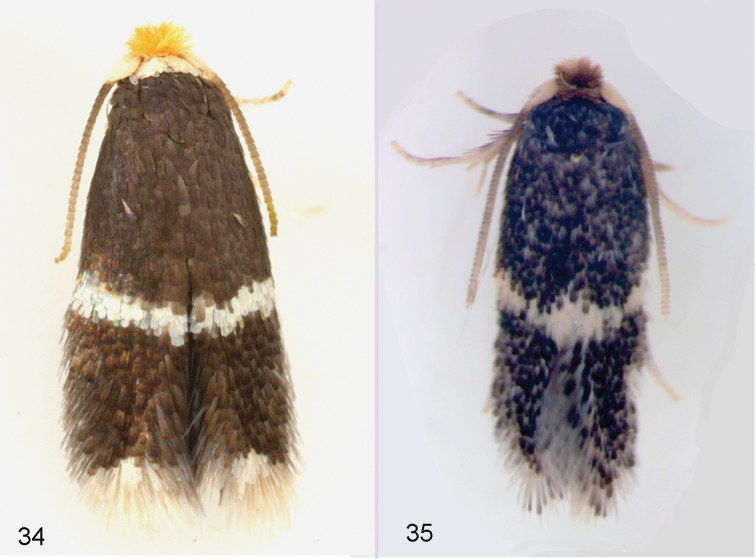
North American *Ulmus* feeding Nepticulidae, adults. **34***Stigmellaapicialbella*, Massachusetts, Hampshire Co., Northampton **35***Ectoedemiaulmella*, male, Tennessee, Blount Co., Townsend. Photographs C. Eiseman, E.J. van Nieukerken.

*Stigmellaapicialbella* (Figure 34) can easily be recognized by the combination of a yellow head, white collar, and forewing with a narrow medial white fascia and an apical triangular white spot extending into the fringe. The basal half of the forewing is a bit more brown or fuscous, whereas the apical part is almost black with coarse scaling. The male genitalia are remarkably “bulky” and do not resemble any other North American species (Newton and Wilkinson 1982).

#### Biology.

*Host plants. Ulmusamericana* L., *U.alata* Michx., *U.rubra* Muhl. (= *fulva* Michx.), *U.thomasii* Sarg. (= *racemosa* D. Thomas), *Ulmus* spp. (Ulmaceae) (Braun 1917, Newton and Wilkinson 1982). *Ulmusalata* constitutes a new host record.

**Figures 36–39.**
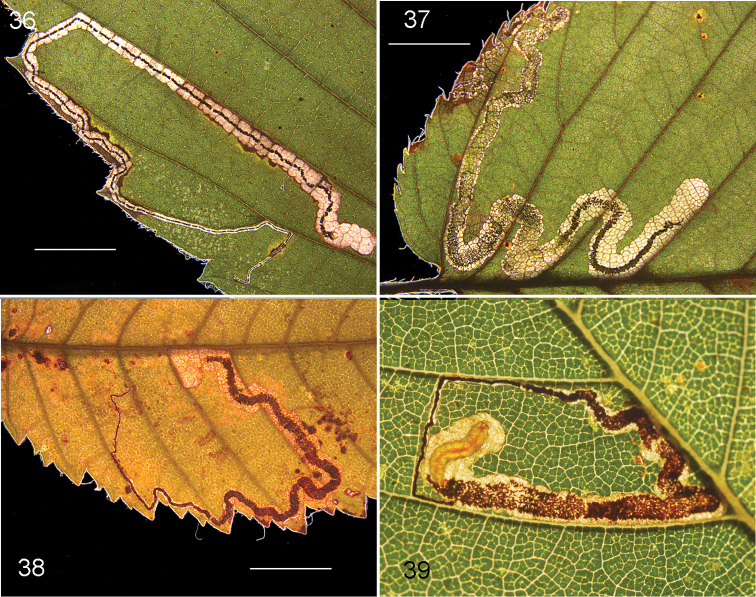
*Stigmellaapicialbella*, leafmines. **36** Vacated mine, Canada, Québec, Brome-Missisquoi, RMNH.INS.40376 **37** Vacated mine, USA, Connecticut, Litchfield Co., Canaan, RMNH.INS.43576 **38** Vacated mine, USA, Mississippi, Oktibbeha Co., Black Prairie Reserve, RMNH.INS.43158 **39** Mine with larva, USA, Tennessee, Obion Co. Scale bars 5 mm. Photographs E.J. van Nieukerken, C. Eiseman (39).

*Leafmine* (Figs 36–39). Egg on either leaf surface, may be against a vein, but never in leaf axil. Mines linear, usually rather straight, partly following veins, or more contorted; frass variable, from narrow linear to contorted, green or brown, sometimes completely filling the mine. Larval exit on leaf upperside.

*Larva* (Figure 39). Yellow, feeding with dorsum upwards; head capsule brown. Larva spinning a brown cocoon on debris.

*Life history.* Bivoltine, or possibly trivoltine (Braun 1917). Larvae in June to early July, again in August to October. Adults recorded from April to early July and again in August.

#### Distribution.

Widespread in Eastern North America, positive records from: Canada: New Brunswick, Ontario (BOLD: BIOUG33718-A12), Quebec (van Nieukerken 2018), USA: Alabama*, Connecticut*, Georgia*, Illinois, Indiana, Kentucky (Chambers 1873), Massachusetts, Mississippi*, New York*, North Carolina, Ohio (Braun 1917), Tennessee, Vermont. States without reference are new records: from states with asterisk we have as yet only seen vacated mines; from the other states the occurrence is confirmed by adults or DNA barcodes of larvae.

#### DNA barcodes.

We have three DNA barcodes, all with BINBOLD: ACG9146 (Figure 44).

#### Remarks.

The leafmines of this species are remarkably variable. Since we have not seen any adults or DNA barcodes yet from the southernmost states, the possibility that some of these mines represent other taxa cannot be excluded.

#### Material examined.

**Canada**: 1 larva, several mines, Québec, Brome-Missisquoi, St Armand, Étang Streit, 7.ix.2015, *Ulmusamericana*; 1♀, Québec, Gatineau, Aylmer, 18 rue Washington, 19.v.1998. **United States**: several vacated mines, Alabama, Monroe Co., Haines Island Park, along Alabama River, 12.x.2010, *Ulmus*; several vacated mines, Connecticut, Litchfield Co., Canaan, Page Road near Falls Village, 11.ix.2011, *U.americana*; 8 vacated mines, Georgia, Murray Co., Chattahoochee Nat. Forest, E of Chatsworth, GA rd 52, 14.x.2010, *U.alata*; 1♂, Indiana, St. Joseph Co., 25.v.2010; 1♂, ibidem, 13.viii.2010; 3♂, ibidem, 15.viii.2010; 1♂, Kentucky [Kenton Co., Covington], Lectotype; 1 adult, Massachusetts, Hampshire Co., Northampton, Northampton Bikeway west of King St., 13.ix.2013, *Ulmus*, emerged 22.v.2014; 3 vacated mines, Mississippi, Oktibbeha Co., Black Prairie Reserve, nr 16^th^ Section Rd, 6.x.2010, *U.alata*; 1 vacated mine, Mississippi, Winston Co., Tombigbee Nat. Forest, Noxubee Hills trailhead, 7.x.2010, *U.alata*; 2 vacated mines, New York, Essex Co, S Wilmington, W branch Ausable river, 13.ix.2011, *U.americana*; 1 larva, mines, Tennessee, Obion Co., Reelfoot Lake, 17.xi.2012, *Ulmus*; 1 larva, 2 mines, Vermont, Addison Co., Addison, Dead Creek WMA, 16.ix.2011, *U.americana*; 1 vacated mine, Vermont, Chittenden Co., Burlington, Colchester Bog, 5.ix.2015, *U.americana*.

*Online photographs*: **Canada**: vacated mine, New Brunswick, York Co., Fredericton, 28.viii.2015, Christopher Adam, https://bugguide.net/node/view/1162297, **United States**: 1 adult, Illinois, Cook Co., Glencoe, 22.v.2017, James F. Steffen, https://bugguide.net/node/view/1373817; mine with larva, North Carolina, Durham Co., Durham, Pelham Road, 25.vi.2016, *U.alata*, Tracy S. Feldman, https://bugguide.net/node/view/1247382, plus many records of vacated mines from NC.

### Ectoedemia ulmella

Taxon classificationAnimaliaLepidopteraNepticulidae

(Braun) Wilkinson & Scoble

Figures 35 , 40–43

Nepticula ulmella Braun, 1912: 87.
Ectoedemia ulmella : Wilkinson and Scoble 1979: 91.

#### Diagnosis.

*Ectoedemiaulmella* (Figure 35) can easily be distinguished from the *Ulmus*-feeding *Stigmella* spp. by the small collar with hair-scales only (*Stigmella* spp. have lamellar scales), combined with the medial white fascia and pale fringe. Other species of *Ectoedemia* may look similar, but males of *E.ulmella* stand out by the brown androconial scales on hindwing upperside. For genitalia see Wilkinson and Newton (1981).

#### Biology.

*Host plants. Ulmusamericana* L., *U.alata* Michx., *U.rubra* Muhl. (= *fulva* Michx.), *U.thomasii* Sarg. (= *racemosa* D. Thomas) (Ulmaceae) (Braun 1917, Wilkinson and Newton 1981). *Ulmusalata* constitutes a new host record.

**Figures 40–43.**
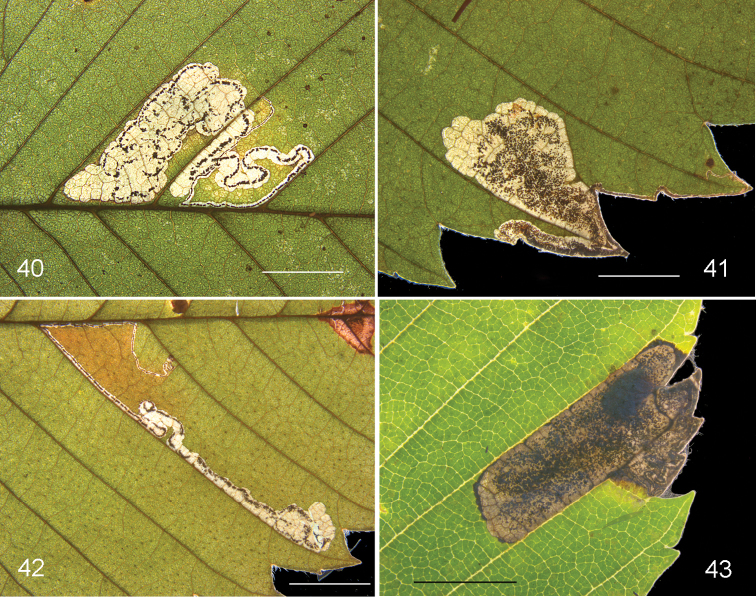
*Ectoedemiaulmella*, leafmines. **40, 41** Vacated mines, Canada, Québec, Brome-Missisquoi, RMNH.INS.40373 **42** mine, larva taken out, barcoded, USA, New York, Essex Co, S Wilmington, larva RMNH.INS.18563. 43 Mine with cocoon, RMNH.INS.43636. Scale bars: 5 mm. Photographs E.J. van Nieukerken.

*Leafmine* (Figs 40–43). Egg usually on upper leaf surface, often against a vein. Early mine a narrow linear tract with broken narrow linear frass, sometimes filled with frass, at first often winding, then straighter, often following a vein, later mine abruptly widening into an irregular blotch with scattered frass. Larval exit on leaf upper side, or cocoon spun inside mine, often in center of blotch (Braun 1917) (Figure 43).

*Larva*. Pale yellowish white, feeding with venter upwards, ganglia usually obvious; head capsule translucent brown. Larva spinning brown cocoon inside mine or on debris.

*Life history*. Larvae found from July to early October, possibly in two generations, but it is also possible that this represents one extended generation. Adults recorded from May to August. From larvae collected in August, the adults emerged the following year.

#### Distribution.

Widespread in Eastern North America, positive records from: Canada: New Brunswick*, Ontario, Quebec, USA: Alabama*, Florida*, Kentucky, Maine, Maryland*, Massachusetts*, Mississippi*, New York, North Carolina*, Ohio, Pennsylvania, Tennessee*, Vermont* (Braun 1917, Wilkinson 1981, Wilkinson and Newton 1981, asterisks indicate new records).

#### DNA barcodes.

**Figure 44.**
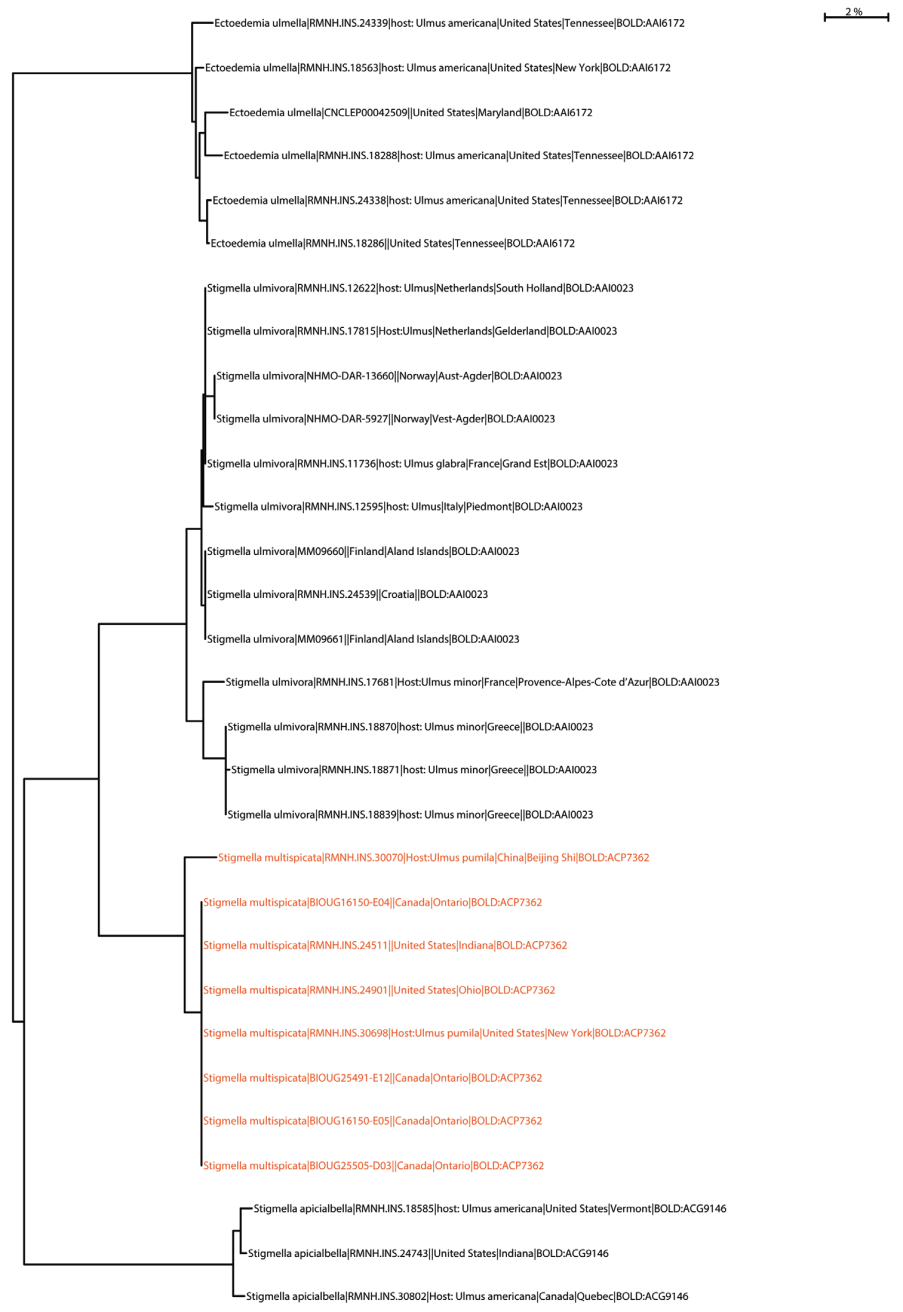
Neighbor Joining tree of DNA barcodes of the treated species. The labels mention: Taxon name, Sample id (collection registry), hostplant (when available), Country, State or Province, BIN number.

We have six DNA barcodes, all with BINBOLD: AAJ6172 (Figure 44).

#### Remarks.

In BOLD there are two specimens from Texas with a distant but related DNA barcode (BOLD:ABX4315); we have not yet checked whether these also belong to *E.ulmella*.

#### Material examined.

**Canada**: 2♂, 1♀, 1 leafmine, Ontario, La Passe, e.l. 22.ii–26.iii.1971, *Ulmusamericana*; 4 leafmines, Ontario, Ottawa, 28.vii–7.viii.1955, *U.americana*; 1♀, 5 leafmines, Ontario, Overbrook, 22.vii–7.viii.1955, *U.americana*; 2 larvae, several mines, Québec, Brome-Missisquoi, St Armand, Étang Streit, 7.ix.2015, *U.americana*; 4 vacated mines, Québec, Gatineau, Aylmer, Deschênes, Ottawa rivershore, 12.ix.2015, *U.rubra*; 1♂, Québec, Gatineau, Aylmer, 11.vi.1989; 1♀, ibidem, 7.vii.1989; 1♂, Québec, Gatineau, Aylmer, 48 rue Notre-Dame, 1.viii.1997; 1♂, 1♀, Québec, Gatineau, Aylmer, 18 rue Washington, 21–22.vi.1999; 3♂, 4♀, 11 leafmines, Québec, Kingsmere, 10–11.ix.1955, e.l. 12.v–17.vii.1956, *U.rubra*; 5 leafmines, Québec, Kingsmere, 1971, *U.americana*; 4 leafmines, Québec, Quyon, 1969, *U.americana*. **United States**: several vacated mines, Alabama, Monroe Co., Haines Island Park, along Alabama River, 12.x.2010, *Ulmus*; several mines, Florida, Volusia Co., Lake Woodruff NWR, Mud Lake Road, 24.ix.2016, *U.americana*; 1♀, Maryland, Calvert Co., Calvert, Scientists Cliffs, 2179 Bluebell Road, 3.viii.2007; vacated mines, Massachusetts, Hampshire Co., Northampton, Northampton Bikeway west of King St., 13.ix.2013, *Ulmus*; several vacated mines, Mississippi, Oktibbeha Co., Black Prairie Reserve, nr 16^th^ Section Rd, 6.x.2010, *U.alata*; 1 larva, several mines, New York, Essex Co., S Wilmington, W branch Ausable river, 13.ix.2011, *U.americana*; several mines, New York, Essex Co, Wilsboro, Noblewood Park, 14.ix.2011, *U.americana*; 1♂, New York, Tompkins Co., Cornell Univ., e.l. 14.viii.1994, *Ulmus*; 3 vacated mines, North Carolina, Haywood Co., NP Great Smoky Mts, Big Creek area, 28.ix.2010, *U.alata*; 3 larvae, several mines, Tennessee, Blount Co., NP Great Smoky Mts, Rich Mountain Gap, 2.x.2010, *U.americana*; 1♂, 2♀, several mines, Tennessee, Blount Co., Townsend, Laurel Valley, 3.x.2010, e.l. 11–19.iv.2011, *U.americana*; 1 vacated mine, Vermont, Addison Co., Addison, Dead Creek WMA, 16.ix.2011, *U.americana*; 2 vacated mines, Vermont, Chittenden Co., Burlington, Colchester Bog, C. Eiseman & J. Blyth, 5.ix.2015, *U.americana*.

*Online photographs*: **Canada**: vacated mines, New Brunswick, York Co., Fredericton (Lincoln Trail), 9.ix.2014, Christopher Adam, https://bugguide.net/node/view/1042642.

## Discussion

Morphology and DNA barcoding show clearly that the North American and Asian populations of *Stigmellamultispicata* are conspecific, suggesting a recent invasion by this species. We think that the invasion must have been from Asia to North America and not the other way around. Although the North American fauna is not yet completely known, a species that is now so abundant and widespread would have been hard to miss by earlier collectors, but not a single older specimen has been found in collections. Also its occurrence on the introduced Siberian elm and (so far) absence from native elm species speaks in favor of an Asian alien. The close relationship between *S.multispicata* and the western Palearctic *S.ulmivora* in the Palearctic *S.ulmivora* species group (van Nieukerken et al. 2016), further supports its Palearctic origin, even though we have no older records than the empty leafmines collected in Beijing in 1984. The DNA barcoding results show that the North American population is slightly different from the single barcode from China: Beijing. This could mean that the source for the invasion is somewhere else in East Asia.

The date of introduction is difficult to assess. Our oldest North American records are from 2010, but since those already are numerous, the species must have been well established several years before that. Since nepticulids are able to spin their cocoons on many substrates, including tree trunks, branches or other objects, we assume that an introduction of larvae or pupae inside cocoons with plants of Siberian elm, or even another plant or object, is most likely; transport of insect invaders with live plant material is considered a major pathway for exotic herbivores (Liebhold et al. 2012, Brockerhoff and Liebhold 2017). Current global traffic has resulted in several recent examples of introductions of leafminers from North America into Europe and vice versa (van Nieukerken et al. 2012b, 2012d, Landry et al. 2013). As far as we know this is only the second instance of a leafmining moth from Asia introduced into North America, the other instance being *Caloptiliatriadicae* Davis, 2013 on the Chinese Tallow Tree *Triadicasebifera* (L.) (Davis et al. 2013). We suspect that the apparent rapid spread of *S.multispicata* may have been aided by the nursery trade. A quick internet search found nurseries in Illinois and Tennessee that will ship Siberian elm plants almost anywhere in the US; although phytosanitary certificates are typically required by states receiving nursery stock, inspection of plants may be less common after they arrive at their destination and even careful inspection of potted or bare-root plants could easily miss the small cocoons. It was recently shown that trade amongst tree nurseries throughout Europe contributed to the spread of the citrus longhorn beetle, *Anoplophorachinensis* (Forster, 1771) (Eschen et al. 2015).

There is a parallel with another invasive Asian leafminer, the weevil *Orchestessteppensis* Korotyaev, 2016 (Korotyaev 2016), also a leafminer of Siberian elm. The species was first found in North America around 2003, then misidentified as the European *O.alni* (Linnaeus, 1758) (Anderson et al. 2007, Looney et al. 2012), and has since spread widely over the United States, including the west (Looney et al. 2012). Also in Asia this species has probably been spreading, and is reported as a local pest species in China (Li et al. 2017). It is possible that *S.multispicata* is also more widespread in Asia, but overlooked due to the limited number of people studying lepidopterous leafminers, and the fact that the mines in small numbers are inconspicuous.

The Siberian elm was introduced in the United States around 1860 and is widely planted for windbreaks and lumber, and now itself an invasive species, particularly in pastures, roadsides and prairies throughout the Midwest and Great Plains regions (Swearingen and Bargeron 2016). It is uncertain whether *S.multispicata* also will colonize American elm species; the American species of *Ulmus* are in a phylogenetically different subgenus, *Oreoptelea* (Spach) Planchon, whereas all Palearctic species belong to subgenus Ulmus L. (Wiegrefe et al. 1994). Larvae of the Siberian Elm specialist *Orchestessteppensis* have not yet been confirmed on American elm species (Looney et al. 2012), but adults have been found feeding on *U.americana* (Anderson et al. 2007), and in Ottawa in 2018 we observed some *Orchestes* mines on *U.americana* in association with abundant *O.steppensis* mines on *U.pumila*. Another invasive Palearctic leafminer, the tenthredinid *Fenusaulmi* Sundevall, 1844, has been recently noted to colonize the indigenous North American species of *Ulmus* (Anonymous 2015).

At the time of writing, *Stigmellamultispicata* already is widespread in eastern North America, from eastern New York to western Iowa, and from Minnesota and Québec to Tennessee. Although the collected material gives a good insight already, online observation websites have been very helpful in providing a quick survey of the distribution, as this source alone was responsible for five of the state records. So far, *S.multispicata* does not appear to have risen to the status of a damaging pest. Although Siberian elm is no longer widely planted as an amenity tree, many specimens remain in urban and residential areas, where the descending larvae and aesthetic damage could be a concern. At this point the damage by *S.multispicata* is local, and probably not yet a great problem. In most places, the mines of *O.steppensis* outnumber those of *S.multispicata*. We advise to follow a prudent course and monitor both species simultaneously to document their spread and impact.

## Supplementary Material

XML Treatment for Stigmella multispicata

XML Treatment for Stigmella ulmivora

XML Treatment for Stigmella apicialbella

XML Treatment for Ectoedemia ulmella

## Supplementary materials

Supplementary material 1 Specimen data of *Stigmellamultispicata* and other *Ulmus* mining Nepticulidae Data type: Microsoft Excel (xlsx) Explanation note: The 288 records are the records of specimens examined and records obtained from online sources, of the taxa treated in this paper and supplement the presented Material examined. Records are listed alphabetically by Species, Country, State, Province, AdminDivision2, AdminDivision3, Locality. Records contain BOLD Process ID’s and GenBank accession numbers. This is an open access article distributed under the terms of the Creative Commons Attribution License (CC BY 4.0), which permits unrestricted use, distribution, and reproduction in any medium, provided the original author and source are credited.Erik J. van Nieukerken, Daniel Owen Gilrein, Charles S. Eiseman
